# Genetic Aberrations in Imatinib-Resistant Dermatofibrosarcoma Protuberans Revealed by Whole Genome Sequencing

**DOI:** 10.1371/journal.pone.0069752

**Published:** 2013-07-29

**Authors:** Jung Yong Hong, Xiao Liu, Mao Mao, Miao Li, Dong Il Choi, Shin Woo Kang, Jeeyun Lee, Yoon La Choi

**Affiliations:** 1 Division of Hematology-Oncology, Department of Medicine, Samsung Medical Center, Sungkyunkwan University School of Medicine, Seoul, Korea; 2 BGI-Shenzhen, Shenzhen, China; 3 Pfizer Oncology, San Diego, California, United States of America; 4 Department of Biology, Copenhagen University, Copenhagen, Denmark; 5 Department of Radiology, Samsung Medical Center, Sungkyunkwan University School of Medicine, Seoul, Korea; 6 Department of Mathematics, Korea University, Seoul, Korea; 7 Department of Pathology, Samsung Medical Center, Sungkyunkwan University School of Medicine, Seoul, Korea; Ohio State University Medical Center, United States of America

## Abstract

Dermatofibrosarcoma protuberans (DFSP) is a very rare soft tissue sarcoma. DFSP often reveals a specific chromosome translocation, t(17;22)(q22;q13), which results in the fusion of collagen 1 alpha 1 (*COL1A1*) gene and platelet-derived growth factor-B (*PDGFB*) gene. The COL1A1-PDGFB fusion protein activates the PDGFB receptor and resultant constitutive activation of PDGFR receptor is essential in the pathogenesis of DFSP. Thus, blocking PDGFR receptor activation with imatinib has shown promising activity in the treatment of advanced and metastatic DFSP. Despite the success with targeted agents in cancers, acquired drug resistance eventually occurs. Here, we tried to identify potential drug resistance mechanisms against imatinib in a 46-year old female with DFSP who initially responded well to imatinib but suffered rapid disease progression. We performed whole-genome sequencing of both pre-treatment and post-treatment tumor tissue to identify the mutational events associated with imatinib resistance. No significant copy number alterations, insertion, and deletions were identified during imatinib treatment. Of note, we identified newly emerged 8 non-synonymous somatic mutations of the genes (*ACAP2*, *CARD10*, *KIAA0556*, *PAAQR7*, *PPP1R39*, *SAFB2*, *STARD9*, and *ZFYVE9*) in the imatinib-resistant tumor tissue. This study revealed diverse possible candidate mechanisms by which imatinib resistance to PDGFRB inhibition may arise in DFSP, and highlights the usefulness of whole-genome sequencing in identifying drug resistance mechanisms and in pursuing genome-directed, personalized anti-cancer therapy.

## Introduction

Dermatofibrosarcoma protuberans (DFSP) is a very rare tumor, with an incidence of only 0.8 to 4.5 cases per million persons per year in the United States [1]–[3]. The standard treatment for DFSP is local surgical excision with wide resection margins [4], [5]. However, local recurrence rates are high because of its infiltrative growth, and there is a small but definite risk (1% to 4%) of distant metastasis [4], [6].

Cytogenetic analysis of DFSP often reveals a specific chromosome translocation, t(17;22)(q22;q13), which results in the fusion of collagen 1 alpha 1 (*COL1A1*) gene and platelet-derived growth factor-B (*PDGFB*) gene [7]–[10]. The resulting COL1A1-PDGFB fusion protein eventually activates the PDGFB receptor (PDGFRB), which acts as a protein tyrosine kinase and a potent growth factor [11], [12]. This discovery prompted further studies on the use of PDGFRB tyrosine kinase inhibitors, such as imatinib, for the treatment of advanced and metastatic DFSP. Imatinib showed good activity against DFSP in preclinical studies and in a series of clinical trials each involving only a small numbers of patients because of the rarity of the disease [11]–[17]. However, a recent pooled analysis of two phase II trials also reported promising clinical activity of imatinib with an objective response rate approaching 50% and a 1-year overall survival (OS) rate of 87.5% [18]. Beyond this however, there is no established salvage treatment after failure of imatinib for DFSP, and the acquisition of imatinib resistance has never been investigated by systematic approaches. Here, we described the results of a whole genome sequencing study on both primary DFSP tumor and tumor cells taken at the time of disease progression after imatinib therapy had failed. We were able to identify a number of genetic changes associated with imatinib resistance.

## Materials and Methods

### Ethics Statement

The protocol was approved by the Samsung Medical Center Institutional Review Board (SMC IRB), and the study was conducted in accordance with the 1996 Declaration of Helsinki. Written informed consent was obtained from the patient.

### Sample Preparation and Whole Genome Amplification

Tumor specimens were collected from a paraspinal mass before and after imatinib treatment by means of computed tomography (CT)-guided biopsy. DNA was extracted from both tumor samples using a commercially available kit. The DNA yield from the post-imatinib tumor was less than 100 ng, and in order to obtain sufficient DNA for library construction, whole genome amplification was performed on approximately 20 ng of this DNA using the REPLI-g Mini Kit (Qiagen) according to the manufacturer's instructions. A 2.5 µL aliquot of template DNA was denatured at room temperature for 3 min and was then neutralized using an acidic buffer provided by the manufacturer. The denatured DNA was mixed with φ29 DNA polymerase and reaction buffer to a total volume of 50 µL and incubated at 30°C for 16 hrs in a GeneAmp PCR System 9700 thermal cycler (Life Technologies). After amplification, the DNA polymerase was inactivated by heating the sample for 3 min at 65°C. In order to increase the yield, three independent reactions were performed and the amplified products were pooled together after whole genome amplification. DNA integrity was evaluated by running the samples on 2% agarose gel electrophoresis. The total yield of DNA was 6.72 µg.

### Library Construction

Libraries of qualified genomic DNA were prepared for paired-end analysis on the Illumina Cluster Station and Illumina HiSeq 2000. To minimize the likelihood of systematic bias in sampling, three paired-end libraries with an insert size of 500 bp were prepared for all samples in this study. The qualified genomic DNA was fragmented into lengths of approximately 500 bp using Covaris (E210). The double-stranded DNA fragments contained 3′ or 5′ overhangs, which were converted into blunt ends using T4 DNA polymerase and Klenow enzyme. Adenosine was added to the 3′ end of the blunt phosphorylated DNA fragments, and adapters were then ligated to both ends. The correctly ligated products were separated by agarose gel electrophoresis and then purified using the QIAquick gel extraction kit. The purpose of this step is to remove residual free and self-ligated adaptors and to select correctly sized templates for cluster generation. DNA fragments with adapter molecules on both ends were selected and amplified. Polymerase chain reaction (PCR) was performed with the two primers that annealed to the ends of the adapters. The number of PCR cycles was minimized to avoid skewing the representation of the library, and the PCR products were checked and purified by agarose gel electrophoresis. They were then used in the library test step and the average fragment size and molar concentration of the library were determined by using an Agilent 2100 Bioanalyzer and an ABI Real-Time PCR System (StepOnePlus™), respectively.

### Sequencing

The sequencing method was based on that of the Illumina HiSeq2000 platform, and we used a fully automated protocol whereby clustered template DNA was sequenced using four-color DNA Sequencing-By-Synthesis technology with reversible terminators and removable fluorescence. Raw image files were processed by the Illumina pipeline for base-calling using the default parameters, and the sequences for each individual were generated as 90 bp paired-end reads. Each library was subjected to 7 lanes, resulting in an at least 50-fold haploid coverage for each sample. The sequencing data were subjected to a strict quality control (QC) test before bioinformatics analysis. The raw whole genome sequence data has been uploaded at the NCBI Sequence Read Achieve (SRA) under the accession number SRA075959.

### Pipeline of Bioinformatics Analysis

The bioinformatics analysis began with the sequencing data (raw data) that was generated from HiSeq2000. In the first step, the adapter sequence in the raw data was removed, and low quality reads that had too many unreadable or low quality bases were discarded, producing “clean data”. For the second step, a *Burrows-Wheeler Aligner* (*BWA*) was used for alignment. *BWA* can generate results in a BAM file format, which is a requirement for a number of the subsequent processes, such as fixing the mate information of the alignment, adding read group information, and removing duplicate reads caused by PCR. After these processes, the final BAM files used for the variant calling were prepared. Single nucleotide polymorphism (SNP) analysis was performed using *SAMtools*, and potential somatic single nucleotide variants (SNVs) were predicted using Varscan (v2.2.5) [19]. We then used our filter pipeline to identify somatic mutations, with the following major criteria: (1) The adjacent somatic mutation distance should be equal to or greater than 10 bp; (2) the mapping quality score should not be significantly lower than 30 (Wilcoxon test, p<0.20); (3) the base quality score should not be significantly lower than 20 (Wilcoxon test, p<0.05); (4) there should be a significant allele frequency change between the tumor and the matched adjacent normal tissue (Fisher's exact test p<0.05); (5) the mutation should not be in gap-aligned reads (less than 10 gap flags within a 20 bp flank region); (6) mutations should not be significantly enriched within 5 bp of the 5′ or 3′ ends of the read (Wilcoxon test, p<0.05); and (7) mutations should not be in a simple repeat region (i.e., the repeat events should be less than 6). Small insertion/deletions (InDels) were detected using *SAMtools*, and structure variants (SVs) and copy number variants (CNVs) were identified using *BreakDancer* and a method we devised by ourselves based on the Segseq algorithm [20], respectively. *ANNOVAR* was used to annotate confident variant results. The final variant scan could then be used in the downstream advanced analysis pipeline. QC was applied throughout the entire process to obtain clean data, alignment and called variants.

### Genomic Profiling

We aligned sequencing reads to the reference genome sequence using the *BWA* software, and these were then mapped using the human genome build 37 (*Hg19*) as the reference genome. The whole genome size of *Hg19* is 3,137,161,264 bp, whereas its effective size is 2,861,327,131 bp (excluding unreadable bases, random regions, hap regions, chromosome Un, and chromosome M in the reference), and is available at http://hgdownload.cse.ucsc.edu/goldenPath/hg19/bigZips/. We used *Picard* software (http://picard.sourceforge.net/) to mark duplication, which is redundant information produced by PCR. After this, the alignment results were merged to give a single BAM format file, which is the compressed binary form of a Sequence Alignment/Map (SAM) file by sample.

The BAM file can be used to visualize aligned reads using *SAMtools* or other genome browsers, such as the *Integrative Genomics Viewer* (*IGV*). For genome re-sequencing, the per-base sequencing depth should theoretically have a Poisson distribution. However, due to the differences between each sample and the reference, the actual distribution might show divergence from the theoretical value. Therefore, this analysis can be used to identify differences between the genome of the sample and the reference. Depth was calculated for each base from the aligned reads. Reads with multiple equal best placements were randomly assigned to one best-hit location. Sequencing depths exhibited a Poisson-like distribution with average depths of 69.70×, 66.55× and 68.26×, for the blood DNA, the imatinib-sensitive (pre-treatment) tumor, and the imatinib-resistant (post-treatment) tumor, respectively.

## Results

### Case History

**Figure 1:**
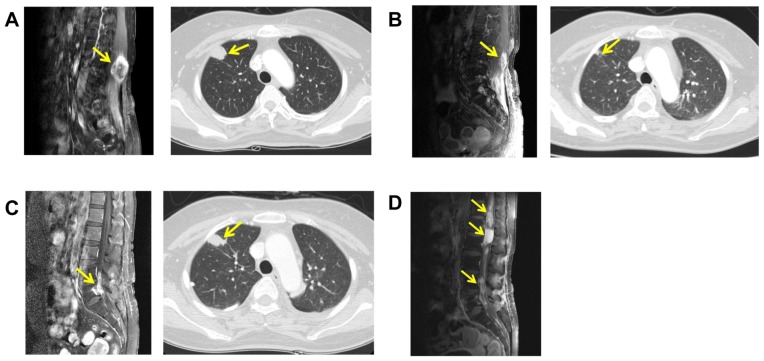
A 46-year old women with DFSP at paravertebral site at the level of L3–4 and RUL zone of chest. Lumbar spine MRI and chest CT were taken. A, before initiation of treatment with imatinib. B, after 4 months treatment with imatinib. C, after further 2 months treatment with imatinib and 3 months treatment with higher dose imatinib. D, at the time of disease progression with leptomeningeal metastasis.

**Figure 2:**
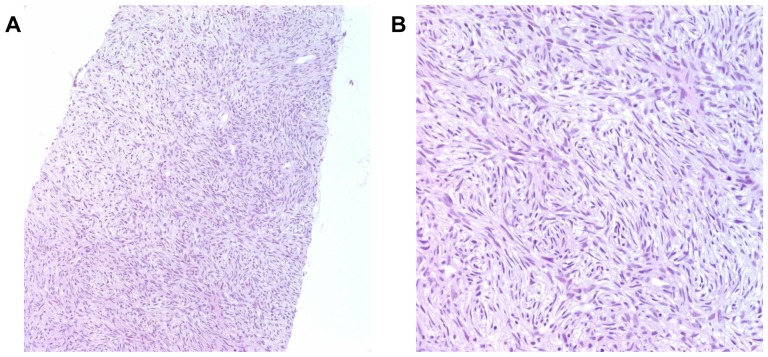
Pathological examination of tumor. A, Hematoxylin-eosin staining is showing spindle-shaped cells organized in a storiform pattern characteristic of DFSP (×100 magnification). B, (×200 magnification).

A 46-year old woman presented at Samsung Medical Center with a paravertebral soft-tissue mass at the level of L3–4. She had been diagnosed four years ago with DFSP that had formed a subcutaneous lesion on the left thigh that was subsequently surgically resected. Three years later, the disease recurred in a paraspinal space at the level of L5–S1, and she had received further surgery followed by postoperative radiotherapy to the surgical bed. At recurrence, she suffered from mild lower back pain and a tingling sensation in the left posterior thigh and the lateral calf area. Magnetic resonance imaging (MRI) of the lumbar spine revealed a 4-cm, lobulated, enhancing soft-tissue mass in the left paravertebral muscle at the level of L3–4 (Fig. 1A; lumbar spine MRI, left panel). A routine chest radiograph showed a nodular lesion in the right upper lung (RUL) zone and a subsequent chest computed tomography (CT) scan showed multiple lung nodules with the largest one located in the RUL zone (Fig. 1A; chest CT, right panel). Core biopsy of left back mass confirmed the presence of metastatic DFSP (Fig. 2), and subsequently, a unique translocation involving chromosomes 17 and 22 was found in the tumor cells (*data not shown*). Hence, the patient was diagnosed with recurrent DFSP with multiple lung metastases that were not amenable to surgical resection.

Accordingly, we started imatinib therapy at a dose of 400 mg per day, every day. After 4 months of this treatment, follow-up spine MRI (Fig. 1B; lumbar spine MRI, left panel) and chest CT (Fig. 1B; chest CT, right panel) confirmed a partial response. After 6 months of imatinib treatment, however, the lung metastases and paraspinal mass were found increased in size, suggesting that the tumors had developed resistance to imatinib. At this point, the dose of imatinib was increased to 800 mg per day, every day. After a further 3 months treatment with this higher dose, the paraspinal mass was found to have grown larger with direct invasion to the L5–S1 neural foramen (Fig. 1C; lumbar spine MRI, left panel). A chest CT scan also showed progressive disease with increased RUL nodule size (Fig. 1C; chest CT, right panel). At this time, after a thorough discussion with the patient and obtaining written informed consent (using the form approved by the SMC Institutional Review Board), we re-biopsied the imatinib-resistant paraspinal mass under CT guidance. The tumor content of the biopsy was found to be over 80% based on pathologic examination by pathologist YRC. DNA was extracted from both this specimen and from the original paraspinal mass, which was sensitive to imatinib, and whole genome sequencing was performed. At this time, the patient was treated with doxorubicin-ifosfamide chemotherapy, sunitinib, and pazopanib, sequentially. The patient did not respond to any of these subsequent regimens and had a rapidly deteriorating clinical course mainly due to progression to leptomeningeal metastases spread from the paraspinal mass (Fig. 1D). Unfortunately, the patient died of disease progression.

### Variation Detection and Annotation

1. **SNP.** We used *SAMtools* to detect SNPs, and *ANNOVAR* for their annotation and classification. A statistical analysis of the SNP distribution (see **supplementary Table 1**) was performed to evaluate the number of SNPs located in different gene regions.
2. **SNV.** Previously, *Varscan* software has mainly been used to identify tumor-specific somatic substitutions by comparing tumor and normal tissue in pairs. Here, we used *Varscan* to identify tumor-specific SNVs by simultaneously comparing read counts, base quality, and allele frequency between the blood/normal tissue (B/N) and the tumor tissue (T) genomes. After identifying the SNVs, we also used *ANNOVAR* for annotation and classification. A statistical analysis of the SNV distribution (see **supplementary Table 2**) was generated to evaluate the number of SNVs located in different gene regions.
  By analyzing the somatic mutation spectrum of each sample, we found that for the normal versus DFSP genomes, G∶C>T∶A accounted for the majority of all detected SNVs. The x-axis denotes the number of SNV mutations, and the y-axis lists each mutation (see **supplementary Fig. 1**). We also analyzed the SNVs in coding sequences and splice regions, and found that, in normal versus tumor genomes, the G∶C>T∶A change was still the most common type of mutation. The x-axis denotes the number of SNV mutations, and the y-axis lists the mutation types (see **supplementary Fig. 2**).
3. **InDel.** We used paired-end reads for gap alignment using *SAMtoolsmpileup* software to detect InDels and *ANNOVAR* to annotate and classify them. A statistical analysis of InDel distribution (see **supplementary Table 3**) was generated in order to evaluate the number of InDels in different gene regions.
  To identify the somatic InDels, those also present in normal samples were filtered out. Hence, we used a program developed in-house to filter the *.vcf files which included the InDel information for normal and tumor samples. A statistical analysis of somatic InDel distribution was generated in order to evaluate the number of InDels in different gene regions.
4. **CNV.** Differences in CNVs between the normal and tumor genomes (see **supplementary Table 4**) were detected by software developed in-house using an algorithm similar to *Segseq* developed by the Broad institute. After identifying the CNVs, we used *ANNOVAR* to annotate and classify them. A statistical analysis of CNV distribution was generated in order to evaluate the number of CNV located in different gene regions.

### Identification of Acquired Gene Aberrations during Imatinib Treatment

**Figure 3:**
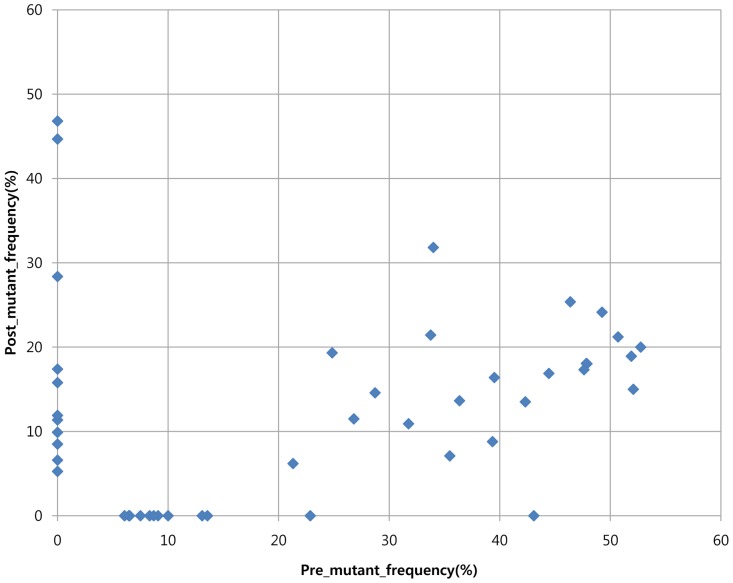
Sequencing data analysis. The value of x-axis denotes the allelle frequency of imatinib-sensitive tumor sample and the value of y-axis denotes the allelle frequency of imatinib-resistant tumor sample of the respective 46 somatic mutations.

In order to identify new mutations upon the emergence of imatinib resistance, we compared the whole genome of the pre- and post-treatment biopsy specimens. Among a total of 46 somatic mutations identified, 22 somatic mutations overlapped between the pre- and post-treatment tumor tissue and 12 somatic mutations were identified only in the pre-treatment tumor tissue (Fig. 3). There were 12 somatic mutations identified only in the post-treatment tumor tissue in which imatinib resistance had developed. Among them, as shown in Table 1, eight non-synonymous mutations were observed in the following genes: *ACAP2*, *CARD10*, *KIAA0556*, *PAQR7*, *PPP1R39*, *SAFB2*, *STARD9*, and *ZFYVE9*. No significant copy number alterations, insertion, and deletions were idenfied during imatinib treatment as shown in supplementary material.

**Table 1:** Newly identified somatic mutations in imatinib-resistant DFSP.

Genes	Genomic change	Amino-acid change	Mutation type	Allele frequency (%) at baseline	Allele frequency (%) at resistance	Gene aberration related to cancer biology	SIFT score	SIFT prediction	COSMIC record
ACAP2	chr3:195041480C>T	Met->Ile	nonsynonymous SNV	0	11.36	NA	0.078	Tolerated	NO
CARD10	chr22:37891880C>G	Glu->Asp	nonsynonymous SNV	0	6.6	YES [47]–[51]	0.674	Tolerated	NO
	chr22:37891912C>G	Asp->His	nonsynonymous SNV				0.002	Damaging	NO
KIAA0556	chr16:27788348G>T	Gly->Cys	nonsynonymous SNV	0	5.26	NA	0.006	Damaging	NO
PAQR7	chr1:26190151G>T	Phe->Leu	nonsynonymous SNV	0	28.37	NA	0.047	Damaging	NO
PPP1R39	chr5:145435750G>A	Arg->Gln	nonsynonymous SNV	0	8.5	YES [52]	0.015	Damaging	NO
SAFB2	chr19:5587776C>T	Gly->Ser	nonsynonymous SNV	0	15.79	YES [53]–[55]	0.016	Damaging	NO
STARD9	chr15:42984506G>A	Gly->Glu	nonsynonymous SNV	0	46.81	YES [56],[57]	0.002	Damaging	NO
ZFYVE9	chr1:52704185G>T	Glu->	stopgain SNV	0	9.89	NA	NA	NA	NO

## Discussion

Using whole-genome sequencing of both pre-treatment and post-treatment tumor tissues, we identified eight non-synonymous and one stop-gain mutations in eight genes that emerged at the same time that the tumor became resistant to imatinib. The accuracy of our 50× sequencing depth and mutation calling method has been validated in another dataset (data not shown) with 82.2% of sensitivity and more than 95% of specificity. This allowed us to identify potential drug resistance mechanisms in this DFSP patient who initially responded well to imatinib but suffered rapidly progressive disease 6 months after treatment. DFSP is a very rare soft tissue sarcoma, and misdiagnosis, multiple equivocal biopsies, and/or a delay in accurate diagnosis are common in the clinical history of the patients with this disease [21]. For a more accurate diagnosis of DFSP, the National Comprehensive Cancer Network guidelines recommend hematoxylin and eosin staining along with immunostaining for markers such as CD34, factor XIIIa, tenascin, and/or stromelysin-3 [22]–[24]. The guidelines do not however recommend re-biopsy when the diagnosis of DFSP is clinically suspicious but not supported by initial pathology [21]. Surgical resection is the standard management approach for localized disease [4], [5], but systemic treatment with imatinib is the standard first-line treatment for inoperable and metastatic DFSP [17], [18], .

Imatinib mesylate (imatinib) is an orally active small-molecule tyrosine kinase inhibitor (TKI) of the breakpoint cluster region-Abelson murine leukemia (BCR-ABL) fusion protein, KIT, platelet-derived growth factor receptor α (PDGFRA), and platelet-derived growth factor receptor β (PDGFRB). Imatinib has revolutionized the treatment of certain malignancies and has become a paradigm for molecular targeted therapy. It has fundamentally changed the way in which chronic myeloid leukemia (CML) is treated, as BCR-ABL is a major driver of this malignancy [25]–[27]. It also shows very high efficacy in the treatment of gastrointestinal stromal tumor (GIST) through targeting KIT and PDGFRA, which have critical roles in oncogenic signaling [28], [29]. Preclinical studies of DFSP showed that the constitutive activation of the PDGFRB tyrosine kinase domain resulting from t(17;22)(q22;q13) translocation together with *COL1A1-PDGFB* gene rearrangement was essential for DFSP pathogenesis [11], suggesting that DFSP could be targeted by imatinib. Subsequently, a number of case reports and a recent pooled analysis of 2 phase II trials (SWOG-S0345 and EORTC 62027) have also reported promising efficacy of imatinib for advanced and metastatic DFSP [13], [14], [17], [18].

Despite this apparent success with imatinib, drug resistance eventually occurs in most CML and GIST cases [30]–[32]. In CML, this can result from further point mutations leading to additional changes in BCR-ABL, for example T315I, Y253H, and F255K [33]. In GIST, secondary KIT mutations in exons 13, 14, or 17 other than exon 11 are reported to be important events in acquired imatinib resistance [34]. When viewed in terms of tumor heterogeneity and clonal evolution, selective therapeutic pressure exerted by imatinib can lead to the elimination of imatinib-sensitive clones and the subsequent expansion of imatinib-resistant clones. As a result, the identification of imatinib resistance mechanisms in CML and GIST could lead to the development of effective second- or third-line treatment strategies, such as imatinib dose escalation and second-generation TKIs including dasatinib and nilotinib for CML [35]–[37] and sunitinib for GIST [38].

Despite these advances, the mechanisms of resistance to PDGFRB inhibition by imatinib and effective second-line treatment strategies for DFSP have not been identified, and DFSP patients who fail imatinib treatment have no further rationale-based treatment options. Recently, next-generation sequencing has enabled us to identify recurrent mutations associated with cancer pathogenesis and to define clonal evolution patterns in diverse malignancies [39]–[44]. Moreover, by comparing mutational changes in the pre-treatment and post-treatment cancer specimens using whole-genome sequencing, important advances in the determination of acquired drug resistance mechanisms to the BRAF inhibitor vemurafenib have been made in malignant melanoma [45], [46].

In this study, we investigated the changes in the mutational spectrum before and after imatinib treatment by whole-genome sequencing and tried to identify the mutational events associated with imatinib resistance in DFSP. The *COL1A1-PDGFB* fusion gene was detected in both imatinib-resistant and imatinib-sensitive tumors and *COL1A1-PDGFB* rearrangement was reconfirmed by PCR, but no point mutation or copy number change was detected in the *PDGFB* gene of the imatinib-resistant tumor. Moreover, most CNVs overlapped between imatinib-resistant and imatinib-sensitive tumors (supplementary material). Taken together, these findings suggest that, although the *COL1A1-PDGFB* fusion gene may have been involved in the original pathogenesis of DFSP, it was not involved the subsequent acquisition of imatinib resistance. Table 1 summarizes eight newly identified mutations in the imatinib-resistant tumor tissue of this DFSP patient, which were not detectable in the imatinib-sensitive tumor. This finding includes mutations in the *CARD10*, *PPP1R39*, *SAFB2*, and *STARD9* genes. *CARD10* is associated with the activation of the NK-kB signaling pathway and is known to have clinical implications in gastric cancer, colon cancer, and non-small cell lung cancer [47]–[51]. A potential role for changes in the *PPP1R39* gene has also been suggested in the development of human cancers [52]. Further, the *SAFB2* gene product is involved in a variety of cellular process, such as cell growth, apoptosis, and stress response and is associated with breast tumorigenesis [53]–[55]. In a recent *in vitro* study, the *STARD9* gene product was shown to be associated with mitotic microtubule formation and cell division and might be a potential candidate target to extend the reach of cancer therapeutics [56]. Among the studies mentioned above, Crone et al. demonstrated that targeting *CARD10* by microRNA-146a inhibited NF-kB signaling pathway activation in gastric cancer cell lines via reduction of tumor-promoting cytokines and growth factors including PDGFRB [49]. This study showed the possible association between *CARD10* inhibition and decreased level of PDGFR and also implied *CARD10* activating mutation may be one of the possible resistance mechanism to PBGFR inhibition by imatinib in DFSP.

We initially undertook this study to identify newly emerged somatic mutations that could help identify salvage treatment strategies specific for this patient. However, despite identifying eight non-synonymous somatic mutations in the imatinib-resistant tumor, there were no drugs or functional studies available for the candidate genes. We plan to prospectively procure more imatinib-resistant specimens of this rare tumor type, which has limited treatment options after the failure of imatinib treatment.

## Conclusions

This study revealed diverse possible candidate mechanisms by which imatinib resistance to PDGFRB inhibition may arise, and suggests the need for further studies for validating *ACAP2*, *CARD10*, *KIAA0556*, *PAAQR7*, *PPP1R39*, *SAFB2*, *STARD9*, and *ZFYVE9* as candidate genes involved in imatinib resistance. The results also highlight the utility of whole-genome sequencing in identifying drug resistance mechanisms and the possibility of genome-directed, personalized anti-cancer therapy based on whole-genome sequencing technology.

## Supporting Information

Figure S1 **Illustration of mutation spectrum for SNV.** The left figure illustrates the total SNV number for each mutation type. C∶G>T∶A and T∶A>C∶G rank the top two categories for mutation number; The right figure illustrates the SNV number for each muation type in CDS and spliced site regions. Fewest SNV in CDS and spliced sites are T∶A>A∶T type. Again, most mutations are C∶G>T∶A. (The X axis indicates different mutation type, and Y axis is the total SNV number in each mutation type category). **Figure S1a.** (a) Illustration of mutation spectrum for SNV in imatinib sensitive DFSP. **Figure S1b.** (b) Illustration of mutation spectrum for SNV in imatinib resistant DFSP.
(DOCX)

Table S1 **Summary table of SNPs.**
(DOCX)

Table S2 **Summary of SNV statistics.**
(DOCX)

Table S3 **Summary of somatic InDels Statistics.**
(DOCX)

Table S4 **Summary of CNV analysis.**
(DOCX)
