# Supplementary figures and images for: Genetic Aberrations in Imatinib-Resistant Dermatofibrosarcoma Protuberans Revealed by Whole Genome Sequencing
Source: PLoS One. 2013 Jul 29;8(7):e69752. doi: 10.1371/journal.pone.0069752 (PMC3726773; doi:10.1371/journal.pone.0069752)

**Figure S1a.**


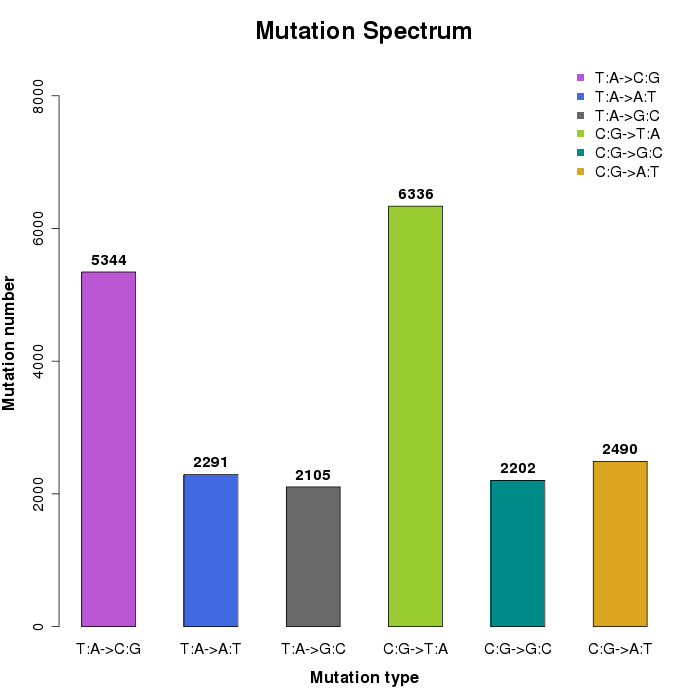

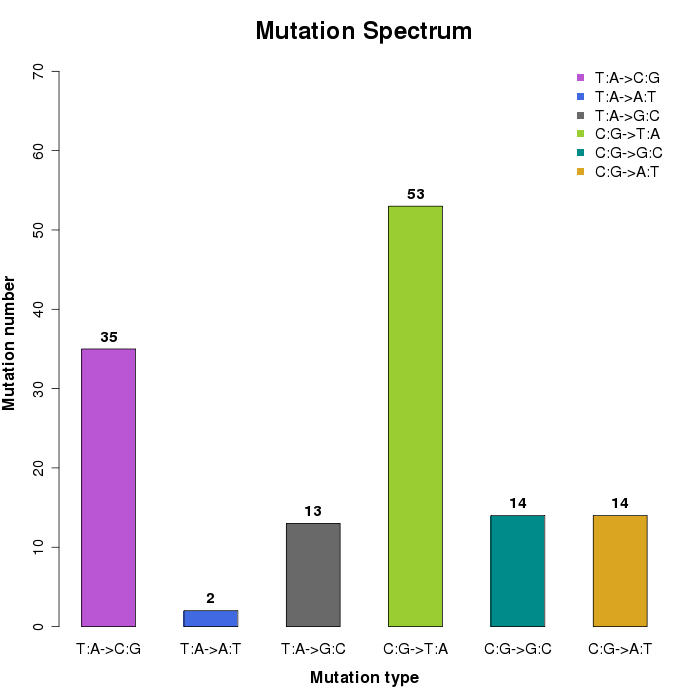


**Figure S1b.**


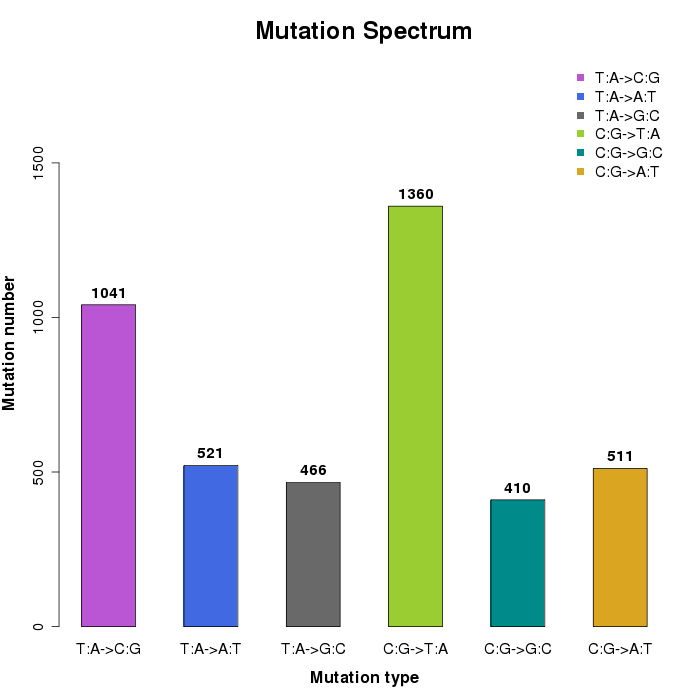

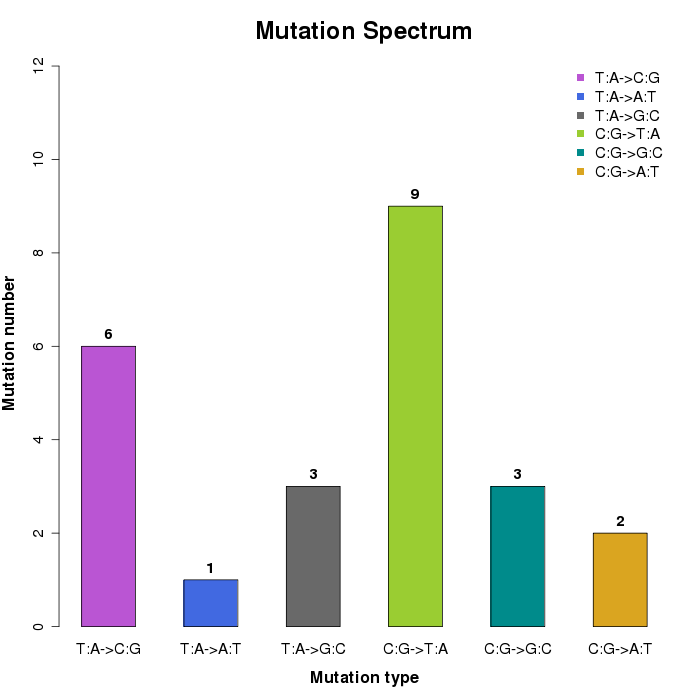

Supplement: Figure S1 — Illustration of mutation spectrum for SNV. The left figure illustrates the total SNV number for each mutation type. C∶G>T∶A and T∶A>C∶G rank the top two categories for mutation number; The right figure illustrates the SNV number for each muation type in CDS and spliced site regions. Fewest SNV in CDS and spliced sites are T∶A>A∶T type. Again, most mutations are C∶G>T∶A. (The X axis indicates different mutation type, and Y axis is the total SNV number in each mutation type category). Figure S1a. (a) Illustration of mutation spectrum for SNV in imatinib sensitive DFSP. Figure S1b. (b) Illustration of mutation spectrum for SNV in imatinib resistant DFSP. (DOCX) [file pone.0069752.s001.docx]
