# Supplementary material for: Genetic Aberrations in Imatinib-Resistant Dermatofibrosarcoma Protuberans Revealed by Whole Genome Sequencing
Source: PLoS One. 2013 Jul 29;8(7):e69752. doi: 10.1371/journal.pone.0069752 (PMC3726773; doi:10.1371/journal.pone.0069752)
Supplement: Table S1 — Summary table of SNPs. (DOCX) [file pone.0069752.s002.docx]

**Table S1.**

| **Categories** | | | **Blood_DNA** | **Pre_treatment** | **Post_WGA** |
| --- | --- | --- | --- | --- | --- |
| Total SNPs | | | 3444587 | 3402902 | 3449174 |
| SNPs in 1000genome and dbsnp132 | | | 3196635 | 3151968 | 3192110 |
| SNPs in 1000genome specific | | | 79122 | 78119 | 79937 |
| SNPs in dbSNP132 specific | | | 94040 | 95788 | 98861 |
| dbSNP rate | | | 95.53% | 95.44% | 95.41% |
| Novel SNPs | | | 74790 | 77027 | 78266 |
| Hom/Het^(1)^ | Hom | | 1483369 | 1528908 | 1482691 |
|  | Het | | 1961218 | 1873994 | 1966483 |
| Function | Exonic^(2)^ | Synonymous | 10119 | 10654 | 10620 |
|  |  | Missense | 9045 | 9517 | 9536 |
|  |  | Stopgain^(3)^ | 77 | 75 | 74 |
|  |  | Stoploss | 30 | 32 | 32 |
|  | Exonic | | 18979 | 19980 | 19969 |
|  | Exonic and Splicing^(4)^ | | 292 | 298 | 293 |
|  | Splicing^(5)^ | | 137 | 150 | 150 |
|  | ncRNA | | 90687 | 90356 | 90824 |
|  | UTR5 | | 3039 | 4469 | 4370 |
|  | UTR5 and UTR3 | | 11 | 11 | 12 |
|  | UTR3 | | 21983 | 21631 | 21995 |
|  | Intron | | 1178871 | 1162961 | 1176211 |
|  | Upstream^(6)^ | | 17238 | 19079 | 18995 |
|  | Upstream and downstream | | 555 | 596 | 603 |
|  | Downstream | | 19734 | 19400 | 19715 |
|  | Intergenic | | 2093061 | 2063971 | 2096037 |
|  | SIFT^(7)^ | | 1173 | 1200 | 1193 |
| Ti/Tv^(8)^ | Ti/Tv | | 2.1164 | 2.1078 | 2.1102 |
|  | dbSNP Ti/Tv | | 2.122 | 2.1145 | 2.1159 |
|  | Novel Ti/Tv | | 1.8791 | 1.8398 | 1.8829 |

Note: The value of the first column takes the following precedence: exonic = splicing >ncRNA>> UTR5/UTR3 > intron > upstream/downstream >intergenic.

1. Hom: homozygous; Het: heterozygous;
2. Exonic here refers only to coding exonic portion, but not UTR portion, as there are two keywords (UTR5, UTR3) that are specifically reserved for UTR annotations.
3. Anonsynonymous SNV, frameshift insertion/deletion, nonframeshift insertion/deletion or block substitution that lead to the immediate creation of stop codon at the variant site. For frameshift mutations, the creation of stop codon downstream of the variant will not be counted as "stopgain".Meanwhile stoploss means that lead to the immediate elimination of stop codon at the variant site.
4. If a variant is located in both Exonic region and Splicing junction, “Exonic and splicing” will be calculated.
5. Splicing in *ANNOVAR* is defined as variant that is within 2-bp away from an exon/intron boundary.
6. The term “Upstream" and "Downstream" is defined as 1-kb away from transcription start site or transcription end site.
7. SIFT(Sorting Tolerant From Intolerant)predicts whether an amino acid substitution affects protein function.
8. Ti refers to Transition, Tv refers to Transvertion.
