# Supplementary material for: Genetic Aberrations in Imatinib-Resistant Dermatofibrosarcoma Protuberans Revealed by Whole Genome Sequencing
Source: PLoS One. 2013 Jul 29;8(7):e69752. doi: 10.1371/journal.pone.0069752 (PMC3726773; doi:10.1371/journal.pone.0069752)
Supplement: Table S2 — Summary of SNV statistics. (DOCX) [file pone.0069752.s003.docx]

**Table S2.**

| Sample | **Blood vs Pre-Tx** | **Blood vs Post-Tx** | **Pre-Tx vs Post-Tx** |
| --- | --- | --- | --- |
| Total | 20,768 | 4,309 | 32,929 |
| 1000genome and dbsnp132 | 2,484 | 445 | 12,157 |
| 1000genome specific | 1,005 | 154 | 1,467 |
| dbSNP132 specific | 5,031 | 1,032 | 5,805 |
| dbSNP rate | 36.19% | 34.28% | 54.55% |
| Novel | 12,248 | 2,678 | 13,500 |
| Hom | 229 | 82 | 362 |
| Het | 20,539 | 4,227 | 32,567 |
| Synonymous | 44 | 10 | 81 |
| Missense | 84 | 14 | 120 |
| Stopgain | 2 | 0 | 1 |
| Stoploss | 0 | 0 | 0 |
| Exonic | 127 | 24 | 202 |
| Exonic and splicing | 3 | 0 | 0 |
| Splicing | 1 | 0 | 4 |
| NcRNA | 863 | 153 | 1178 |
| UTR5 | 9 | 3 | 34 |
| UTR5 and UTR3 | 0 | 0 | 1 |
| UTR3 | 49 | 31 | 179 |
| Intronic | 3,308 | 1,384 | 8,094 |
| Upstream | 101 | 40 | 192 |
| Upstream and downstream | 2 | 0 | 5 |
| Downstream | 116 | 24 | 240 |
| Intergenic | 16,189 | 2,650 | 22,800 |
| SIFT | 21 | 2 | 24 |
| Ti/Tv | 1.2852 | 1.2584 | 1.573 |
| dbSNP Ti/Tv | 1.571 | 1.095 | 1.8785 |
| Novel Ti/Tv | 1.1194 | 1.3368 | 1.2351 |
