# Supplementary material for: Genetic Aberrations in Imatinib-Resistant Dermatofibrosarcoma Protuberans Revealed by Whole Genome Sequencing
Source: PLoS One. 2013 Jul 29;8(7):e69752. doi: 10.1371/journal.pone.0069752 (PMC3726773; doi:10.1371/journal.pone.0069752)
Supplement: Table S3 — Summary of somatic InDels Statistics. (DOCX) [file pone.0069752.s004.docx]

**Table S3.**

| **Categories** | **Blood vs Pre-Tx** | **Bood_vs Post-Tx** | **Pre-Tx vs Post-Tx** |
| --- | --- | --- | --- |
| Total | 7,577 | 5,617 | 7,577 |
| 1000genome and dbsnp132 | 397 | 171 | 397 |
| 1000genome specific | 802 | 687 | 802 |
| dbSNP132 specific | 571 | 402 | 571 |
| dbSNP rate | 12.78% | 10.20% | 12.78% |
| Novel | 5,807 | 4,357 | 5,807 |
| Hom | 7,577 | 5,617 | 7,577 |
| Het | 0 | 0 | 0 |
| Frameshift Insertion | 1 | 0 | 1 |
| Non-frameshift Insertion | 1 | 5 | 1 |
| Frameshift Deletion | 0 | 0 | 0 |
| Non-frameshift Deletion | 1 | 2 | 1 |
| Frameshift block substitution | 0 | 0 | 0 |
| Non-frameshift block substitution | 0 | 0 | 0 |
| Stopgain | 1 | 1 | 1 |
| Stoploss | 0 | 0 | 0 |
| Exonic | 4 | 8 | 4 |
| Exonic and splicing | 0 | 0 | 0 |
| Splicing | 0 | 0 | 0 |
| NcRNA | 185 | 157 | 185 |
| UTR5 | 4 | 8 | 4 |
| UTR5 and UTR3 | 0 | 0 | 0 |
| UTR3 | 41 | 32 | 41 |
| Intronic | 2,566 | 2,120 | 2,566 |
| Upstream | 39 | 56 | 39 |
| Upstream and downstream | 2 | 3 | 2 |
| Downstream | 39 | 41 | 39 |
| Intergenic | 4,697 | 3,192 | 4,697 |
