# Supplementary material for: Genetic Aberrations in Imatinib-Resistant Dermatofibrosarcoma Protuberans Revealed by Whole Genome Sequencing
Source: PLoS One. 2013 Jul 29;8(7):e69752. doi: 10.1371/journal.pone.0069752 (PMC3726773; doi:10.1371/journal.pone.0069752)
Supplement: Table S4 — Summary of CNV analysis. (DOCX) [file pone.0069752.s005.docx]

**Table S4.**

| **Categories** | **Blood vs Pre-Tx** | **Blood vs Post-Tx** | **Pre-Tx vs Post-Tx** |
| --- | --- | --- | --- |
| Total | 123,683 | 160,535 | 296,967 |
| Exonic | 24,880 | 38,134 | 48,116 |
| Exonic and splicing | 0 | 0 | 0 |
| Splicing | 4,343 | 4,687 | 5,573 |
| NcRNA | 3,400 | 4,984 | 8,251 |
| UTR5 | 2 | 0 | 3 |
| UTR5 and UTR3 | 0 | 0 | 0 |
| UTR3 | 179 | 187 | 413 |
| Intronic | 30,172 | 35,148 | 77,439 |
| Upstream | 1,684 | 3,117 | 3,095 |
| Upstream and downstream | 72 | 106 | 87 |
| Downstream | 878 | 1,087 | 1,651 |
| Intergenic | 58,073 | 73,085 | 152,339 |
